# John Snow: The Pioneer of Modern Epidemiology and Anesthesia

**DOI:** 10.7759/cureus.67602

**Published:** 2024-08-23

**Authors:** Christos S Avdulla, Ntaniela Tachirai

**Affiliations:** 1 Department of Public Health, University of Patras, Patras, GRC; 2 Department of Public Health Policy, University of West Attica, Athens, GRC

**Keywords:** public health, cholera, anesthesia, epidemiology, john snow, historical vignette

## Abstract

John Snow (15 March 1813-16 June 1858) stands as a seminal figure in the fields of epidemiology and anesthesia. His groundbreaking work in tracing the source of cholera outbreaks and advancing the practice of anesthesia has left an indelible mark on modern medicine. Born in York, England, Snow's early passion for science and dedication to medical practice led him to become a pioneering force in his field. His meticulous methods, including the innovative use of spatial analysis and statistical mapping, challenged prevailing theories and laid the groundwork for modern public health initiatives. Snow's contributions to anesthesia, particularly his work with ether and chloroform, revolutionized surgical practices, significantly improving patient care and safety. This article delves into Snow's life, achievements, and the innovative processes he employed, underscoring his enduring impact on human health. By examining his legacy, we aim to enhance our understanding of medical history and inspire both present and future healthcare professionals, honoring the legacy of this medical hero.

## Introduction and background

**Figure 1:**
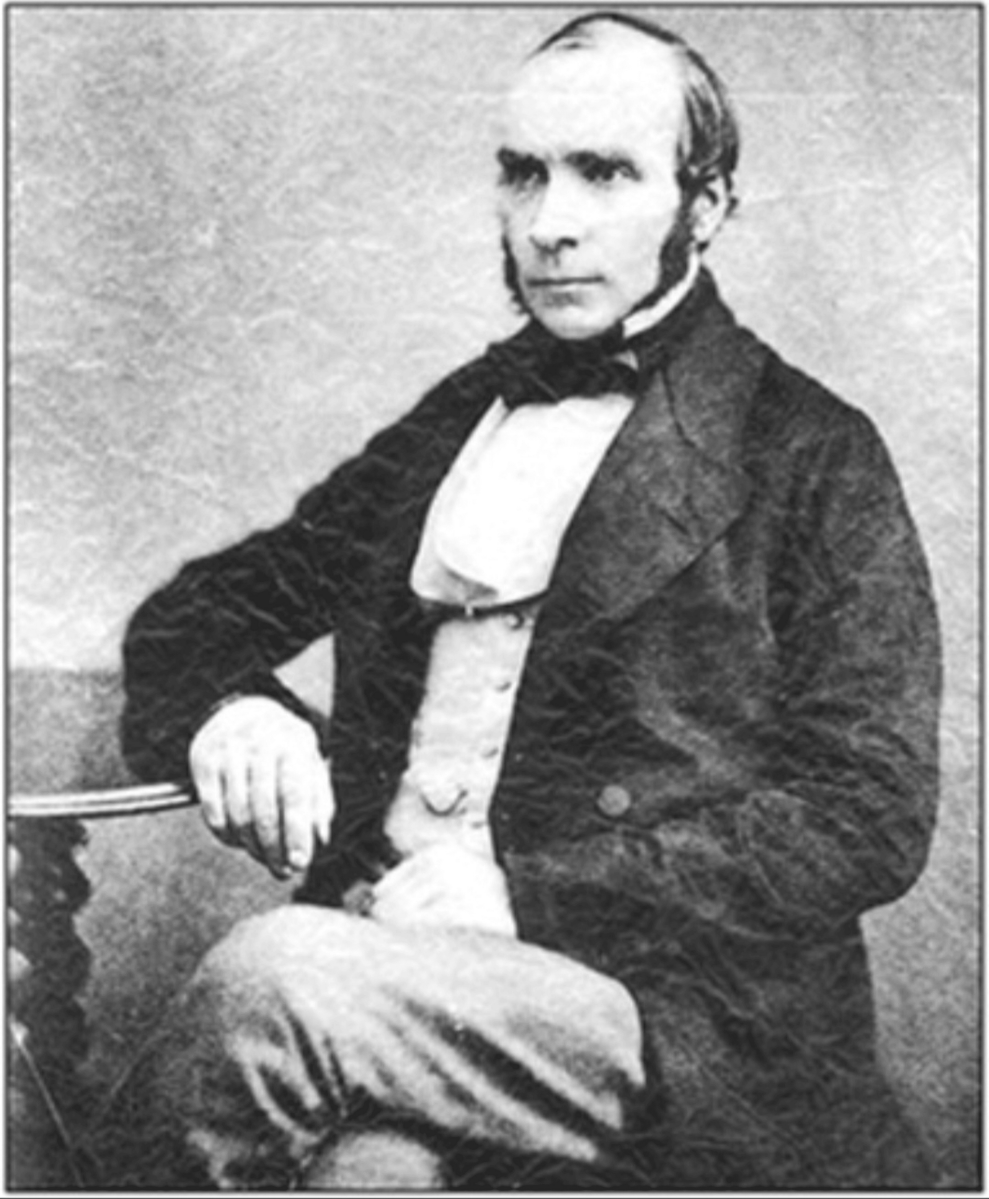
John Snow Source: Library, Archive & Open Research Services blog [2]

John Snow's (Figure 1) name is synonymous with the birth of epidemiology and the development of anesthesia techniques. His meticulous methods and innovative thinking have not only transformed medical practices in his time but also laid the foundation for future advancements in public health [1].

Snow was born on March 15, 1813, in York, England, into a working-class family. His early life was marked by a passion for learning and a dedication to the sciences, despite the financial constraints of his upbringing. At the age of 14, he began an apprenticeship with a surgeon in Newcastle upon Tyne, where he developed his foundational medical skills. This early exposure to clinical practice and the harsh realities of medical treatment without effective pain management significantly influenced his later work in anesthesia [3,4].

Snow's medical education continued in London, where he attended lectures at the Hunterian School of Medicine before enrolling at the University of London. He graduated with an MD in 1844. During his studies, Snow was particularly influenced by the prevailing scientific debates and the emerging fields of medical research and public health, which were gaining prominence during the Victorian era [1,3].

His early medical career was characterized by a combination of clinical practice and research. Snow was an active member of various medical societies, including the Westminster Medical Society, where he presented papers on topics ranging from respiratory diseases to the effects of various gases on the human body. These early experiences and his commitment to empirical research set the stage for his later groundbreaking work in epidemiology and anesthesia [5,6].

The purpose of this article is to explore the life, achievements, and innovative processes of John Snow, underscoring his enduring impact on human health. By delving into his contributions, we aim to enhance our understanding of medical history and inspire both present and future healthcare professionals, honoring the legacy of this medical pioneer.

## Review

Contributions to anesthesia

John Snow's most notable contributions lie in the field of anesthesia, where his pioneering work fundamentally changed the practice of surgery and pain management. During the mid-19th century, surgery was an excruciating and often deadly experience due to the lack of effective pain relief. The introduction of anesthesia marked a turning point, and Snow was at the forefront of this medical revolution [7].

Snow's interest in anesthesia began in 1846, following the demonstration of ether anesthesia in the United States. He quickly recognized the potential of ether and conducted extensive experiments to understand its effects and improve its administration. Snow developed a portable ether inhaler, which allowed for more controlled and safer delivery of the anesthetic agent. His meticulous approach and innovative designs significantly reduced the risks associated with ether anesthesia [1,7].

However, it was his work with chloroform that truly cemented his reputation. Chloroform, introduced by Scottish obstetrician Sir James Young Simpson, quickly gained popularity due to its potency and rapid onset. Snow's contributions to chloroform anesthesia were multifaceted. He conducted systematic studies on its physiological effects, carefully observing the stages of anesthesia and identifying the optimal dosages. His findings were published in several papers and later compiled in his seminal book, "On Chloroform and Other Anaesthetics" (1858), which became a cornerstone in the field of anesthesiology [8].

One of Snow's significant achievements was his administration of chloroform to Queen Victoria during the births of Prince Leopold in 1853 and Princess Beatrice in 1857. This royal endorsement played a crucial role in the widespread acceptance of anesthesia. Snow's careful monitoring and precise administration during these high-profile cases demonstrated the safety and effectiveness of chloroform, alleviating public and professional skepticism [3,4].

Snow's innovations were not limited to the clinical use of anesthetics. He also emphasized the importance of understanding the pharmacokinetics and pharmacodynamics of these agents. His research included detailed observations of the effects of different anesthetic agents on respiration, circulation, and consciousness. Snow's ability to combine clinical practice with rigorous scientific inquiry sets new standards for the medical community [6,8].

Moreover, Snow's invention of the chloroform inhaler, which allowed for controlled and measured doses, was a significant advancement. This device minimized the risks of overdose and ensured consistent administration, significantly improving patient safety. His attention to detail and commitment to patient care were evident in his meticulous approach to anesthesia, which prioritized both efficacy and safety [8,9].

The cholera breakthrough

John Snow's most celebrated achievement is his investigation of the 1854 Broad Street cholera outbreak in London. At a time when the miasma theory (the belief that diseases were caused by "bad air") was predominant, Snow proposed that cholera was waterborne. This theory was initially met with skepticism, as the miasma theory was widely accepted among the medical community [6].

**Figure 2:**
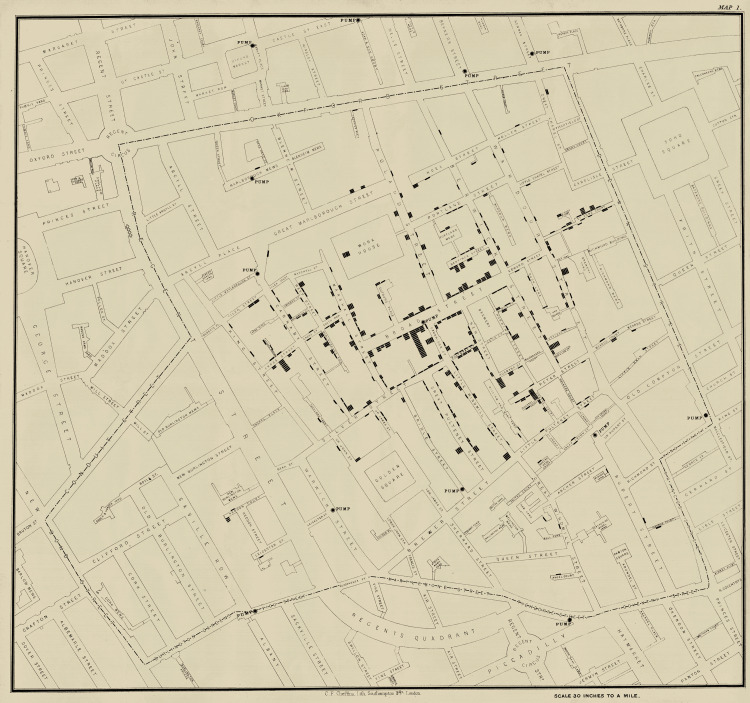
Snow’s cholera map Source: Library, Archive & Open Research Services blog [2]

Snow's groundbreaking approach involved meticulous data collection and analysis. He began by mapping the locations of cholera cases (Figure 2), a novel method at the time, which allowed him to visualize the spread of the disease. This innovative use of spatial analysis revealed a pattern centered around the Broad Street water pump. Snow conducted interviews with local residents, gathering detailed information on their water sources and daily routines. His methodical approach provided strong empirical evidence that linked the outbreak to the contaminated water supply from the pump [1,6].

One of the most compelling pieces of evidence came from the examination of the cases among the workers at the nearby Lion Brewery and the inmates of a workhouse, both of which had their own private water supplies. These groups had significantly lower rates of cholera compared to those using the public pump. This clear correlation between the source of water and the incidence of cholera cases strengthened Snow's argument against the miasma theory [10,11].

Snow's investigation culminated in the removal of the pump handle on Broad Street, a decision made in collaboration with local authorities. This action effectively ended the outbreak, providing a practical demonstration of his theory. The removal of the pump handle is often cited as a defining moment in the history of public health and epidemiology [8,10,11].

Snow's seminal work, "On the Mode of Communication of Cholera" (1855), provided a comprehensive analysis of the patterns of cholera transmission and argued convincingly against the miasma theory. He used statistical data to support his findings, a practice that was pioneering at the time. Snow's detailed maps and case studies in the book are considered among the earliest examples of epidemiological methods that are still in use today [1,8].

Moreover, Snow's work laid the groundwork for the development of modern public health infrastructure. His findings underscored the importance of clean water supplies and proper sewage systems in preventing the spread of infectious diseases. This realization led to significant public health reforms in London and other major cities, ultimately improving urban sanitation and reducing the incidence of waterborne diseases [8,12].

Innovative processes and methods

John Snow's approach to scientific inquiry was characterized by meticulous data collection, spatial analysis, and rigorous hypothesis testing. His innovative use of these methods not only advanced the understanding of cholera transmission but also set new standards for public health research [8,11].

One of Snow's most significant contributions was his use of detailed maps to correlate cholera cases with specific water sources. This method of spatial analysis was revolutionary at the time and is considered one of the earliest examples of geographical information system techniques. By plotting the locations of cholera cases on a map of Soho, London, Snow was able to visually demonstrate the clustering of cases around the Broad Street pump. This visual representation provided compelling evidence that supported his hypothesis of waterborne transmission [8,13].

Snow's map of the 1854 cholera outbreak is now iconic in the field of epidemiology. It illustrated the power of visual data in identifying disease patterns and sources of infection. This approach has since become a fundamental tool in public health, used to track and control outbreaks of various diseases. Snow's pioneering use of spatial data highlighted the importance of location-based analysis in understanding and mitigating public health threats [13].

In addition to his spatial analysis, Snow's meticulous data collection methods were groundbreaking. He conducted thorough investigations by interviewing affected families, collecting detailed demographic information, and examining water sources. This comprehensive approach allowed Snow to gather robust empirical evidence, which he used to construct a persuasive argument for the waterborne transmission of cholera. His systematic data collection and analysis were pioneering steps toward the development of modern epidemiological methods [1,12,13].

Snow also employed innovative statistical techniques to analyze his data. He calculated attack rates and compared them between different groups, such as those who drank water from the contaminated pump versus those who did not. These statistical comparisons provided strong quantitative support for his hypothesis, demonstrating the effectiveness of combining qualitative and quantitative data in medical research [14].

Furthermore, Snow's approach emphasized the importance of interdisciplinary collaboration. He worked closely with local authorities, other medical professionals, and community members to gather data and implement public health measures. This collaborative approach was crucial in removing the pump handle on Broad Street and curbing the cholera outbreak. Snow's work demonstrated the value of combining expertise from various fields to address complex public health challenges [11,13].

Snow's innovative methods extended beyond his work on cholera. His research in anesthesia also showcased his commitment to scientific rigor and innovation. He conducted controlled experiments to understand the effects of anesthetic agents, developed new techniques for their safe administration, and emphasized the importance of precise dosage and patient monitoring. These contributions laid the foundation for modern anesthesia practices and underscored Snow's role as a pioneer in both epidemiology and anesthesiology [1,3,4,8,13].

Enduring impact and legacy

John Snow's contributions to medicine have left an indelible mark on both epidemiology and anesthesia, establishing principles and practices that endure to this day. His work emphasized the critical importance of data-driven decision-making and scientific rigor, setting new standards for public health research and medical practice [15].

Snow's pioneering efforts in epidemiology laid the groundwork for modern public health initiatives. His meticulous methods of data collection, spatial analysis, and hypothesis testing are foundational to contemporary epidemiological studies. The principles he established for investigating disease outbreaks continue to guide public health professionals in managing infectious diseases, from cholera to modern pandemics like COVID-19 [1,16,17]. His use of statistical analysis and geographic mapping to track the source of cholera outbreaks remains a cornerstone of epidemiological methods [13].

In the field of anesthesia, Snow's advancements revolutionized surgical practices and patient care. His development of safer, more effective methods for administering ether and chloroform significantly improved the safety and comfort of surgical patients. The principles he established regarding dosage control and patient monitoring are still fundamental to anesthesia practice today [7,8].

Snow's legacy also extends to his role in advocating for public health reforms. His findings on the waterborne transmission of cholera underscored the necessity of clean water supplies and proper sewage systems, influencing major public health reforms in London and beyond. These reforms played a crucial role in improving urban sanitation and reducing the prevalence of waterborne diseases [8,11,13].

Moreover, Snow's interdisciplinary approach, involving collaboration with local authorities and community engagement, highlighted the importance of collective action in addressing public health challenges. His work demonstrated that effective public health interventions require the combined efforts of medical professionals, policymakers, and the public [1,8,13,18].

## Conclusions

John Snow's life and work exemplify the profound impact that one individual can have on the field of medicine. His pioneering efforts in anesthesia and epidemiology have not only transformed medical practices but have also set enduring standards for scientific investigation and public health. As we honor his legacy, we are reminded of the importance of innovation, perseverance, and the continuous pursuit of knowledge in advancing human health.
